# Magnesium Administration in Preslaughter Drinking Water Reduces Spinal Fractures in Pigs during Stunning

**DOI:** 10.1021/acsomega.5c08255

**Published:** 2025-12-19

**Authors:** Renata V. C. Gabriel, Wander S. F. Filho, Ana Vitória G. de Sousa, Geancarlo G. Degane, Alexandre B. do Amaral, Ednaldo C. Guimarães, João Paulo R. Bueno, Robson Carlos Antunes

**Affiliations:** † 28119 Federal University of Uberlândia (UFU), Uberlândia 38400-694, MG Brazil; ‡ Federal Institute of Goiás, Urutaí Campus, Urutaí 75790-000, GO Brazil

## Abstract

Spinal fractures in pigs during electrical stunning are frequent and compromise animal welfare, meat quality, and generate economic losses. Despite the relevance of the problem, specific preventive strategies are still little explored. This research evaluated, in an unprecedented way, the effect of magnesium supplementation in drinking water during preslaughter fasting on the occurrence of these fractures. A total of 2,080 pigs were evaluated, divided into a control group (CT) with 964 animals, not supplemented and a treatment group (TG) with 1,116 animals, supplemented with magnesium in drinking water, at a concentration of 300 mg/L. The supplementation reduced the proportion of fractures from 39.4% (380/964) to 30.0% (335/1,116), with an odds ratio of 0.66, suggesting a protective effect associated with muscle relaxation. Pigs with fractures had lower water holding capacity (WHC: 0.338 vs 0.351; *p* = 0.0076), especially in the supplemented group (0.333 vs 0.357; *p* = 0.0002), confirming the relationship between skeletal injuries and meat quality. In conclusion, preslaughter supplementation with magnesium is practical and effective to reduce fractures and improve pork quality.

## Graphical abstract

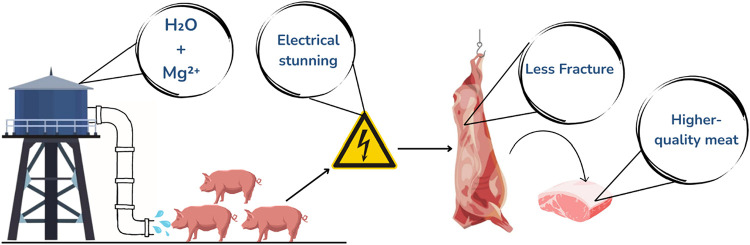

## 1 Introduction

Spinal fractures observed during the electrical stunning of pigs constitute a relevant problem in the production chain: they compromise animal welfare, reduce yield and carcass quality, and cause economic losses due to convictions. Pork has technological attributes—appearance, texture, juiciness, color, fat content, pH, and water holding capacity (WHC)—which are affected by physiological responses to stress in the preslaughter stage. During preslaughter management, handling, transport, and containment situations often trigger acute stress responses and neuromuscular excitement, increasing the chance of adverse events at the time of stunning. The passage of electric current can cause intense muscle contractions that, in extreme cases, result in vertebral fractures, often located in the lumbosacral region.^1–3^

Despite the relevance of the problem, there are few studies that systematically describe the occurrence of vertebral fractures in pigs during electrical stunning,^3,4^ and there are only a limited number of studies that have evaluated the use of magnesium as a strategy to mitigate preslaughter stress—although the results have been varied, most have indicated beneficial effects of supplementation, mainly on meat quality parameters.^5,7^ Alves et al. tested the supplementation of four minerals in the drinking water of pigs at preslaughter and observed that magnesium was the most effective in improving meat quality. However, none of these studies evaluated the occurrence of vertebral fractures.

In this context, magnesium supplementation administered by drinking water during preslaughter fasting emerges as a practical and quickly applied alternative. Previous evidence indicates that magnesium acts as a modulator of the nervous system and muscle contraction, reducing the release of cortisol and catecholamines, providing greater muscle relaxation.^5–7^ These physiological properties justify the hypothesis that the administration of magnesium hours before slaughter can attenuate the high motor response to electric shock and, consequently, reduce the intensity of involuntary contractions responsible for fractures. In addition to that, interventions that reduce preslaughter excitement can have a positive impact on animal welfare and meat technology parameters such as WHC. Studies that applied magnesium for a short period of time reported beneficial effects on stress and WHC, but they are still scarce in the specific context of vertebral fractures during stunning.^8–11^

This research evaluated in a direct and applied way the effect of magnesium supplementation via drinking water during preslaughter fasting on the occurrence of spinal fractures in pigs submitted to electrical stunning. In addition, it was investigated the relationship between the occurrence of fractures and subcutaneous fat thickness, as well as the possible interactions between magnesium supplementation and the water holding capacity (WHC) of meat. This article differs from previous investigations as it tests a short-term intervention, easily adopted in a commercial setting, focused specifically on the occurrence of fractures during electrical stunning. The main expected contribution is to provide practical and direct evidence of a simple strategy to reduce skeletal injuries at slaughter, with immediate implications for animal welfare and for quality and industrial efficiency of pork production.

## 2 Materials and Methods

This research was approved by the ethics committee on the use of animals, process number 23117.017394/2025–90.

### 2.1 Location and Period of Research

The experiment was conducted in May 2025, over six consecutive days, in a slaughterhouse located in the city of Uberlândia, in the Triângulo Mineiro region, Minas Gerais, Brazil; under Municipal Inspection Service. The establishment has an operational capacity for the slaughter of approximately 350 pigs per day, has 16 pigsty stalls and a sequestration stall. Each one has 10 pacifiers to supply water to the animals, containing cement floors, masonry partitions with iron gates and tin roof. The structure of the slaughterhouse’s pens complies with Ordinance N° 274/94, which defines 1m^2^ per pig with an average weight greater than 110 kg in Brazil.

### 2.2 Animals and Experimental Management

A total of 2080 finished pigs from different commercial lines were used, from the following genetic crosses: males AGPIC337 × females TN70; males LQ1250 × females DB90; and males AGPIC337 × Camborough females. All of these commercial lines are crosses originated from males of the Large White, Landrace, Duroc and Pietrain breeds; and females from Landrace and Large White breeds. Half of the animals were of males surgically castrated and the other half were females. The pigs were equally distributed among two experimental groups: control (C) and treatment (T), in order to balance genetic crosses and sex in both groups. All animals had an average weight of 120 kg and an approximate age of 178 days.

The animals were from commercial farms located in the Triângulo Mineiro region, Minas Gerais, Brazil, and were raised in intensive production systems: In conventional warehouse, with natural ventilation and stocking density compatible with the animal welfare recommendations for the finishing phase. The food offered consisted of a balanced commercial diet, provided until the beginning of the preslaughter fasting.

Solid fasting was started 18 h before slaughter, according to operational protocols established between the farm and the slaughterhouse. The transport to the slaughter site was carried out by trucks, and the average travel time was less than 2 h. The boarding and landing stages were conducted by trained workers aiming to minimize stress and preserve the animal’s welfare.

### 2.3 Experimental Design and Treatments

At the slaughterhouse, the animals of this research (*n* = 2080) were divided into two experimental groups: control (C; *n* = 964, 482 males and 482 females) and treatment (T; *n* = 1,116, 558 males and 558 females). The allocation was made in a systematic way, positioning the groups on opposite sides of the pigsty, in order that one side was destined to the control group (without the addition of magnesium sulfate in the drinking water) and the other to the treated group (with the addition of magnesium sulfate in the drinking water). They arrived at the slaughterhouse pigsties approximately 12 h before slaughter, and had free access to water from the moment of arrival.

To supply drinking water to the animals, two water tanks of 1000 L each were allocated. For the control group the water pacifiers were supplied from the water tank containing 1000 L of drinking water without magnesium sulfate supplementation, and for the treatment group, it was supplied from the water tank containing 1000 L of drinking water with magnesium sulfate supplementation.

The magnesium dosage used in the experiment was defined based on the recommendations of the National Research Council (NRC, 2005), which establishes 300 ppm (or 300 mg/L of water) as the maximum tolerated level of magnesium for pigs, as described by Alves (2011). To ensure the safety and efficacy of supplementation, an inorganic source of the mineral was chosen, whose concentration of magnesium available after dissolution in water corresponds to 9.10%. In that case, the amount offered to the animals was calculated in order to reach the desired concentration in drinking water, using 3296 kg of the supplement for every 1000 L of water. This strategy allowed the dose to be adjusted to the recommended safe limit, optimizing supplementation and avoiding possible adverse effects resulting from excess mineral. The animals in the treated group consumed the supplemented water for a period of 10 to 12 h before slaughter. The water consumption per group did not exceed 700 L during this preslaughter period.

Before the beginning of the experiment, water samples were collected from the pigsties where the research was conducted, aiming to define its mineral composition, especially the pre-existing concentration of magnesium. The analysis of chemical compounds in the water indicated an average magnesium content of approximately 80 ± 2 ppm, and it was carried out at the Chemical Analysis Center (CEAQ) at the Federal University of Uberlândia (UFU) by the method of flame atomic absorption spectrometry.

### 2.4 Data Collection

#### 2.4.1 Animal Behavior Assessment over Preslaughter

The behavioral analyses were all visual, monitored by the same evaluators, during the preslaughter time. Over the rest period it was observed that the environment remained calm and with no records of agitation events. The nocturnal vocalizations were discrete and compatible with the expected behavior for finishing pigs, without the occurrence of collective stress. The lights remained on throughout the night, favoring the observation of the animals.

The treated group showed predominantly passive behavior, remaining lying down most of the time, getting up only to consume water. Both groups remained visibly clean, with no accumulation of dirt or signs of discomfort.

The animals were taken to the slaughter line sequentially, stall by stall, and four pigs were released at a time. Three employees of the slaughterhouse, wearing standardized blue uniforms, were responsible for handling the animals, followed by a member of the research team who observed and recorded the process. The movement occurred calmly and continuously, without interruptions or the use of excessive stimuli. Before entering the stunning box, all the animals went through a shower, according to the routine practice of the slaughterhouse.

The climatic conditions during the 6 days of the experiment were obtained from the meteorological database of the National Institute of Meteorology (INMET), referring to the station of Uberlândia, Minas Gerais. The average air temperature was 22.3 °C, with minimum and maximum values of 21.8 and 22.8 °C, respectively. The relative humidity of the air averaged 62.1%, with variations between 60.1% (minimum) and 64.2% (maximum). The average dew point temperature was 14.4 °C. These conditions characterize a thermally comfortable environment for finishing pigs, favoring rest and reducing the risk of heat stress during the preslaughter fasting period.

#### 2.4.2 Fractures Assessment, Backfat Thickness Measurement, and *Longissimus Dorsi Muscle* Sample Collection

The swine was stunned by means of electric shock equipment, using two electrodes positioned in the cephalic region and a third electrode applied in the cardiac region. The operational parameters used were: voltage of 366 V and current of 1.6 A at the cephalic electrodes, and current of 1.1 A at the cardiac point, with a frequency of 60 Hz. The time of application of the electrical stimulus was seven seconds. The mean interval between stunning and the beginning of bleeding ranged from five to eight seconds. Bleeding was conducted by cutting the carotid artery, with an average duration of 3 min, according to the routine of the slaughterhouse.

After bleeding, the carcasses were submitted to the usual stages of the slaughter process, starting with scalding in tanks with water heated between 62 and 65 °C, in order to facilitate the pelage removal. Then, they went through epilator machines equipped with rotating cylinders for the mechanical elimination of hair and dirt from the skin. After waxing, the carcasses were subjected to direct flame in a blowtorch to burn residual hair, followed by manual scraping (toilet). After that it was performed an evisceration opening the abdominal cavity and removing the thoracic and abdominal viscera for post-mortem inspection, according to current legal requirements.

After the post-mortem inspection stage, the occurrence of spinal fractures was identified by a member of the research team through direct visual inspection of the carcass, based on the observation of blood clots and lesions compatible with microfractures in the spinal region. Examples of carcasses with fractures are illustrated in Figure 1.

**Figure 1:**
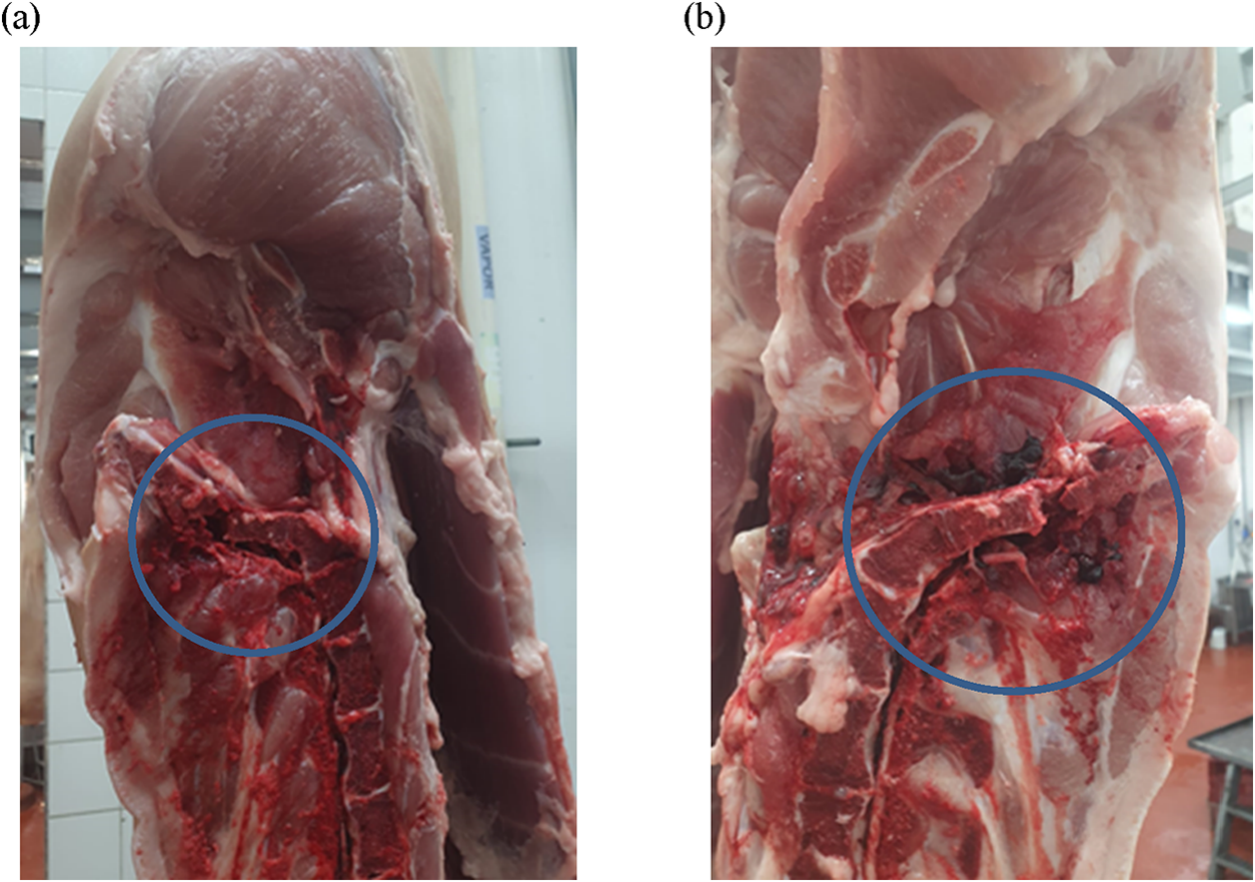
Examples of pig carcasses with fractures: (a) fracture in the lumbosacral region; (b) fracture with the presence of blood clots. Photos from the authors’ personal archive.

The carcasses with and without fractures were recorded in a schedule, according to the number previously marked on the left palette of the animals. This method was adopted because it is the only viable way to identify fractures in real time within the slaughter line flow without interrupting the industrial process, in addition to being commonly used in field evaluations due to its speed and practical applicability.

Subcutaneous fat thickness was measured in all carcasses immediately after their longitudinal division. The measurement was performed with the aid of a millimeter flexible ruler, positioned perpendicular to the surface of the carcass, at a point located on the loin, at the height of the last rib, always on the left half carcass. This method was chosen because it is a direct, practical and widely used technique in slaughterhouses for the evaluation of carcass conformation, allowing standardization of measurements. All measurements were recorded in a schedule before the carcasses were entered for cooling, by the same previously trained observer.

Then, the carcasses were washed with potable water and sent for cooling in cold rooms, kept between 0 and 4 °C. After the cooling period, which means reaching a temperature ≤7 °C, the carcasses with fractures and without fractures were located once again by the number recorded on the left shoulder and a sample of the *Longissimus dorsi muscle* of approximately 2 cm^2^ was collected from each previously selected carcass using a scalpel and sterile anatomical forceps, always from the left half carcass. The data collection was carried out by a member of the research group.

The samples were packed in plastic tubes with lids, identified with the carcass number and the day of slaughter, and transported in a thermal box containing gel ice and kept refrigerated until the end of the analyses, which occurred on the same day of collection. Collections were carried out at the same time, ensuring a sample of the carcass with fracture and another of a carcass without fracture for each group.

The identification of the experimental group each sample belonged control (C; *n* = 964) or treatment (T; *n* = 1116) was performed retrospectively, based on the count of animals allocated to each stall and the order of entry into the slaughter line. As the groups were kept on opposite sides of the pigsty and conducted sequentially to slaughter, it was possible to establish the correspondence between the carcass number (previously recorded on the left shoulder) and the respective group of origin. This strategy allowed the traceability of the samples without interfering in the operational routine of the slaughterhouse and ensured the correct classification of the data according to the experimental treatment.

#### 2.4.3 Water Holding Capacity (WHC) Assessment

At the Meat Technology Laboratory of the Federal University of Uberlândia (UFU), the samples remained refrigerated (between 0 and 4 °C) until the WHC analyses were performed on the same day of collection. The determination of the water holding capacity (WHC) was performed using the compression method, a widely recognized technique considered the gold standard for this evaluation, as described by Grau and Hamm^11^ and adopted by several recent studies.^12^ The steps of the procedure are illustrated in Figure 2.

**Figure 2:**
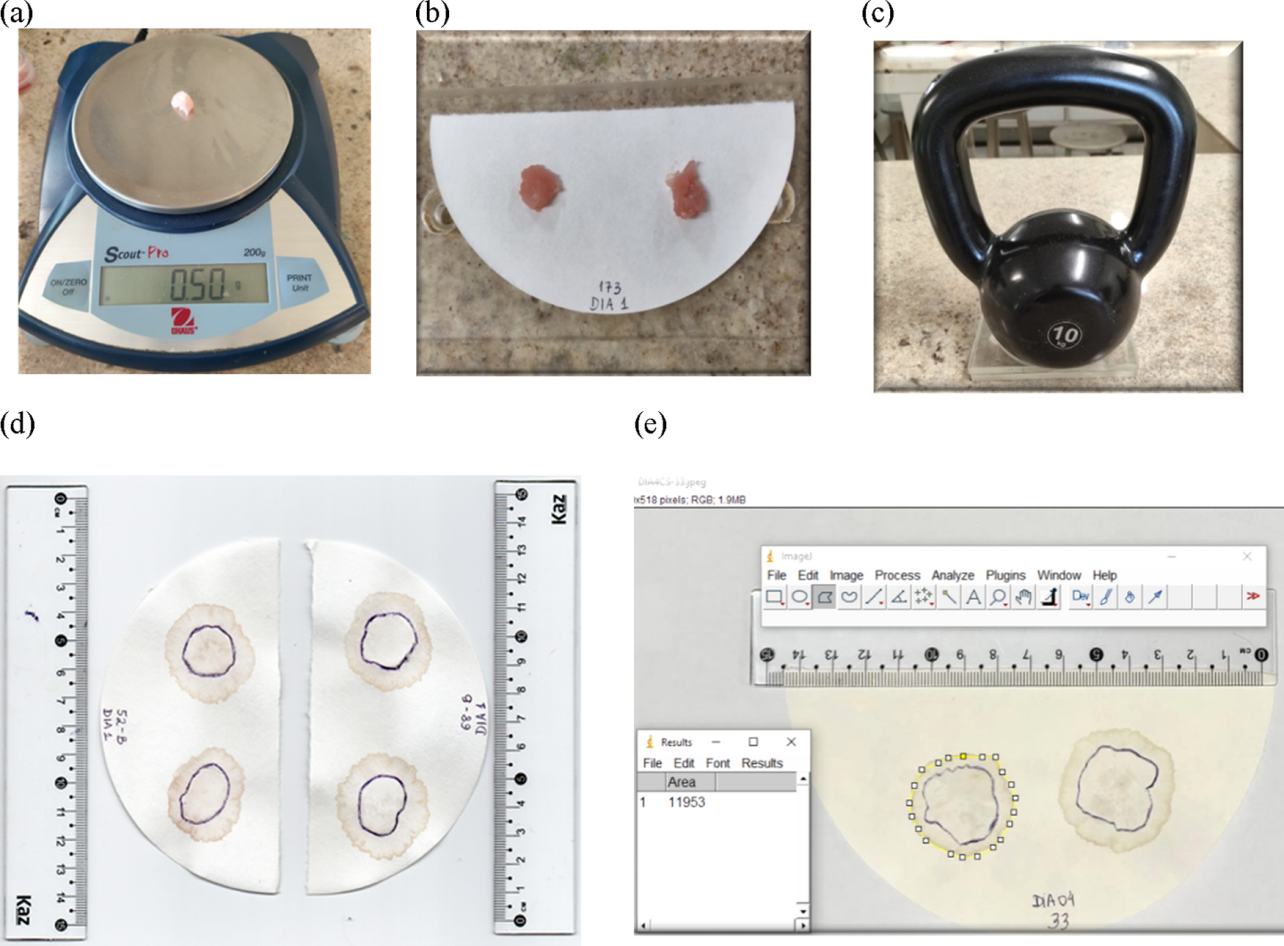
Stages of the water holding capacity (WHC) analysis: (a) standardization of the weight of the samples; (b) *Longissimus dorsi* sample on filter paper; (c) compression method with standardized weight; (d) image of the postcompression sample; (e) analysis of WHC images in the *ImageJ software*. Photos from the authors’ personal archive.

The analysis of the water holding capacity (WHC) was performed in 495 samples of *the Longissimus dorsi muscle* of pigs. They were conducted in duplicate, totaling 990 determinations. For each sample, the mean of the values obtained in the duplicates was considered as representative of the WHC. The determination of the results was expressed by the ratio between the pressed meat area and the total area of the impression, indicating the capacity of the sample to hold water under pressure.

The samples were standardized in portions of 0.5 g and weighed individually on a precision scale (OHAUS Scout Pro). For each pair, a quantitative round filter paper (Unifil brand, medium filtration, white color, diameter of 15 cm) was used, duly identified with the carcass number (animal ID) and the date of collection. The two samples were placed side by side on the filter paper and compressed between two acrylic plates, on which a standardized weight of 10 kg was applied for 5 min.

After this period, the weights and the top paper were removed simultaneously, and the filter paper showed the formation of two distinct areas: one corresponding to the pressed meat and the other to the exudate halo. This area of the meat was outlined on the back of the paper with a marker. Afterward, the papers were photographed and the images analyzed using the *ImageJ* v. 1.37 software on which the areas of pressed meat and exudate were accurately calculated. The results were expressed in the form of ratios between the areas, and the average values obtained from the two samples of each carcass were entered and organized in Microsoft Excel 2010 schedule for later statistical analysis.

### 2.5 Statistical Analysis

Statistical analysis was conducted to assess if the proportion of fractures differed between the control (C; *n* = 964) or treatment (T; *n* = 1116) groups, and to verify the relationship between the occurrence of fractures and subcutaneous fat thickness, as well as the possible association between water holding capacity (WHC) and magnesium use. The comparison between the proportions of fractures in groups C and T was performed using the binomial test to compare two proportions, adopting a significance level of 5% (*p* < 0.05). The association between categorical variables, such as group and presence of fracture, was analyzed using the odds ratio (OR), considering group C as reference 1 (one) and group T as 0 (zero); fracture as 1 (one) and absence of fracture as 0 (zero). The OR values were followed by the respective 95% confidence intervals (CI), and were considered statistically significant when the CI limits did not include value 1.

For the quantitative variable subcutaneous fat thickness, the normality and homogeneity of the residues were initially verified using the Shapiro-Wilk and Levene tests, both with a significance level of 5%. As the data did not attended the assumptions required for analysis of variance (ANOVA), after data transformation, it was decided that the application of the nonparametric Kruskal–Wallis test. The analysis of the WHC was also performed by calculating the odds ratio (OR), considering the same group and fracture codings, with a CI of 95%.

All statistical analyses were performed using the R software (R Core Team, 2025), version 4.3.1, using the statistical programming language and environment of the R Foundation for Statistical Computing, Vienna, Austria. Available at: https://www.R-project.org.

## 3 Results

### 3.1 Occurrence of Vertebral Fractures in Pigs

The group (C) consisted of 964 pigs had 380 that suffered fractures and 584 that did not suffer fractures whereas group (T) consisted of 1,116 pigs had 335 with fractures and 781 that did not suffer fractures. The binomial test for two proportions (odds ratio–OR) indicated that the incidence of fractures was significantly higher in the control group (39.4%) compared to the treated group (30.0%) (*p* = 0.000008; 95%CI: 5.2%–13.6%). The odds ratio analysis indicated that pigs supplemented with magnesium had a lower probability of fractures, with an OR of 0.66 (95%CI: 0.55–0.79), which corresponds to a 52% reduction in the chance of fracture compared to the animals in the control group.

### 3.2 Association between Fracture Occurrence, Experimental Group and Subcutaneous Fat Thickness

Subcutaneous fat thickness was significantly associated with the occurrence of fractures. Pigs with fractures had a lower average thickness of subcutaneous fat (1.98 cm), while those without fractures had an average of 2.14 cm. The odds ratio analyses showed that a 1 cm increase in backfat thickness was associated with a 72% reduction in the chance of fractures (OR = 0.58). When stratified by group, this reduction was 82% in the Treatment group (OR = 0.55) and 49% in the Control group (OR = 0.67). It was confirmed a statistical significance of the odds ratios for the occurrence of fractures, both as a function of the experimental group and of the thickness of subcutaneous fat, as 95% confidence intervals did not include a value of 1.

These findings indicate a real effect of these variables on the probability of fractures in pigs. The animal in group (T) has a 52% lower chance of presenting the fracture.

Based on the results of the Kruskal–Wallis test, Table 1 presents the descriptive values of subcutaneous fat thickness (cm), stratified by experimental group (C) and (T) and the occurrence or absence of fractures.

**Table 1:** Average Thickness of Subcutaneous Fat (cm) in Pigs with and without Fractures, According to the Experimental Group^a^,^b^

	average thickness (cm)	
	Fracture	No fracture
group	CS; *n* = 380; TS; *n* = 335	CN; *n* = 584; TN; *n* = 781
Control (C; *n* = 964)	1.96^bA^	2.07^aB^
Treatment (T; *n* = 1116)	2.00^bA^	2.18^aA^

a Source: the authors, 2025.

b Different lowercase letters (a, b) on the line indicate a significant difference between the presence and absence of fracture (*p* < 0.05). Different capital letters (A, B) in the column indicate a significant difference between groups (*p* < 0.05).

The analysis stratified by experimental group revealed that pigs with fractures had significantly lower subcutaneous fat thickness than those without fractures. In group (C), the fractured animals had an average of 1.96^b^ cm, while the nonfractured animals had 2.07^a^ cm. Similarly, in the group (T), the thickness was 2.01^b^ cm in the animals with fractures and 2.18^a^ cm in those without fractures. These results confirm a significant association between lower subcutaneous fat thickness and the occurrence of fractures in both groups (*p* < 0.05).

When comparing the experimental groups considering only the animals with fractures (“Fracture” column), there was no significant difference in the mean thickness between the Control (1.96^A^ cm) and Treatment (2.01^A^ cm) groups.

In general, the Kruskal–Wallis test indicated a highly significant association between subcutaneous fat thickness and the occurrence of fractures, regardless the experimental group (*p* = 1.29 × 10^–11^).

### 3.3 Water Holding Capacity (WHC)

The WHC was calculated by the ratio between the area of the compressed meat and the total area of the spot (meat + exudate). Higher values indicate less exudate release and, therefore, greater water holding capacity by the meat. WHC was significantly associated with the occurrence of fractures—animals without fractures had higher WHC. The animals were divided into two experimental groups: control (C; *n* = 238), subdivided into CS (*n* = 119, animals with fractures) and CN (*n* = 119, animals without fractures), and treatment (T; *n* = 257), subdivided into TS (*n* = 127, animals with fractures) and TN (*n* = 130, animals without fractures).

The odds ratio for the group (0 = Treatment; 1 = Control) was 1.47 (95%CI: 0.06–35.76), indicating no statistically significant association with WHC. However, the occurrence of fractures was strongly associated with WHC: the OR was 0.015 (95%CI: 0.0005–0.3962), suggesting that for each 1 percentage unit increase in WHC, the chance of fracture was about 66.6 times lower.

When analyzing only the group (T), the association between WHC and fracture was even more evident: the OR was 0.00015 (95%CI: 0.00000103–0.0212), indicating a strong reduction in the chance of fracture with increasing WHC. Considering increments of 1 percentage unit in the WHC, the chance of fracture was approximately 6,667 times lower for each increase. In group (C), there was no statistically significant association between WHC and fractures.

The Kruskal–Wallis test did not identify a statistically significant difference in the (WHC) between groups (C) and (T) in general (*p* = 0.30). However, when considering the occurrence of fractures, it was observed that pigs without fractures had significantly higher WHC than those with fractures, regardless of the experimental group (*p* = 0.0076), with medians of approximately 34.5 and 33.5, respectively. These results suggest an association between carcass integrity and greater water holding capacity.

The analysis stratified by experimental group revealed that this association was especially evident in the group (T), where the animals without fractures had significantly higher WHC than the fractured group (*p* = 0.0002). Among the pigs in group (T), the average WHC was 35.71 for those without fracture and 33.32 for those with fractures. In group (C), there was no significant difference between the animals with 34.39 and without fractures 34.43.

When comparing the Control and Treatment groups within the “no fracture” condition, it was found that the pigs in group (T) had significantly higher WHC than those in group (C) (*p* = 0.0126). This indicates that magnesium supplementation may have contributed positively to water holding in animals that did not suffer fractures. On the other hand, among the animals with fractures, no significant differences in WHC were observed between the experimental groups (*p* = 0.24) Table 2.

**Table 2:** Water Holding Capacity (WHC) in Pigs with and without Fractures, According to the Experimental Group^a^,^b^

	ratio of compressed meat area to total spot (meat + exudate)	
group	fracture	no fracture
Control	34.39^bA^	34.43^aB^
C; *n* = 238, CS; *n* = 119, animals with fractures and CN; *n* = 119, animals without fractures.		
Treatment	33.32^bA^	35.71^aA^
T; *n* = 257; TS; *n* = 127, animals with fractures and TN; *n* = 130, animals without fractures.		

a Source: the authors, 2025.

b Different lowercase letters (a, b) on the same line indicate a significant difference between pigs with and without fractures within the same group. (*p* < 0.05). Different capital letters (A, B) in the column indicate significant difference between groups for the same fracture condition (*p* < 0.05; Kruskal–Wallis test). WHC = water holding capacity expressed by the ratio of the area of the compressed meat to the total area of the spot (meat + exudate).

The Water Holding Capacity (WHC) of the meat was significantly different between pigs with and without fractures, regardless of the experimental group. In both groups, animals without fractures had higher WHC than those with fractures (*p* < 0.05). In addition, when comparing the experimental groups in the “nonfracture” condition, it was observed that the pigs in the group (T) had significantly higher WHC than those in the group (C) (35.71 vs 34.43; *p* = 0.0126). This finding suggests that magnesium supplementation may exert an indirect effect on the improvement of WHC, possibly by reducing the occurrence of fractures and, consequently, the preslaughter stress that affects meat quality. No difference between the groups was observed between the animals with fractures.

## 4 Discussion

### 4.1 The Effect of Magnesium Supplementation on the Occurrence of Fractures

The magnesium supplementation via drinking water provided during the preslaughter period was effective in reducing the occurrence of fractures in pigs when compared to the control group. Unlike dietary supplementation, this approach allows for rapid action in fasting animals, making it a practical alternative for critical moments of preslaughter management.^5^

The effects observed can be attributed to magnesium’s properties as a stress modulator and muscle relaxant.^5,15^ The supplementation possibly reduced neuromuscular excitability and modulated the behavior of the animals, making them less reactive to external stimuli and sudden movements during electrical stunning—factors directly associated with the risk of vertebral fractures. The action of magnesium occurs, in part, due to its calcium antagonist effect, which stabilizes the cell membrane and promotes muscle relaxation, as demonstrated in previous studies.^14–16^

Although physiological indicators of stress were not measured in this research, results of systematic reviews indicate that magnesium supplementation in pigs can reduce plasma cortisol levels, improve stress-related behaviors, and reduce the occurrence of traumatic injuries.^5,6,13^ Furthermore, the effect of magnesium may be influenced by other minerals present in the diet, such as calcium and phosphorus, which compete for absorption and modulate muscle excitability and bone strength, although these interactions were not evaluated in this research.

### 4.2 Relationship between Subcutaneous Fat Thickness and Fracture Occurrence

The results of the relationship between the occurrence of fractures and the thickness of subcutaneous fat showed that this characteristic can influence the susceptibility to bone lesions during the process of electrical stunning in pig slaughter. The fractured animals had thinner back fat thickness. This significant association between subcutaneous fat thickness and the occurrence of fractures (OR = 0.58 for each additional cm) reinforces the hypothesis that a thicker fat layer can provide mechanical protection to bones at slaughter, especially in the spinal region.

A one-cm increase in subcutaneous fat thickness was associated with up to a 72% reduction in the chance of fracture, regardless of the experimental group. The association observed between greater thickness of subcutaneous fat and lower occurrence of fractures suggests that subcutaneous fat may play a mechanical protective role during stunning at slaughter.

Anatomical differences among pigs can make them more or less susceptible to vertebral fractures,^2^ which reinforces the existence of individual factors involved in this injury. According to Hu et al. (2023), in a research with Duroc pigs, subcutaneous fat thickness was positively correlated with bone mineral density, indicating that animals with greater fat deposition had denser bones and, potentially, were more resistant to fractures. These findings suggest that anatomical and body composition characteristics—such as fat thickness and bone quality—may practice a protective structural role against mechanical impacts during handling and slaughter. However, electronarcosis remains the main trigger for these fractures, and losses in meat and carcass quality are inevitable, regardless of the type of equipment, the configurations used, or the training of operators.^17^

Pigs with the current genetics are genetically improved and tend to have less subcutaneous fat deposition and higher lean meat yield. Consequently, they have less fat coverage and a higher proportion of muscle mass. This characteristic can directly influence the response to electrical stunning, since more developed muscles, with less fat insulation, tend to present more intense contractions during the passage of electric current.^2^

This intensity of response can increase the risk of vertebral fractures, especially in the absence of precise electrode placement or when there are individual variations in anatomy. In view of this scenario, the hypothesis considered is that the electrical stunning parameters currently used (voltage and amperage), developed based on previous genotypes, may not be the most appropriate for the zootechnical profile of modern pigs. Therefore, additional studies evaluating the interaction between animal biotype and electronarcosis parameters are required, aiming to minimize fracture losses and improving welfare at slaughter.

Although studies directly related to the production of commercial pigs are scarce, these findings reinforce the hypothesis that the thickness of subcutaneous fat at the level of the last rib can act as a natural shock absorber, minimizing the effects of impacts and sudden movements during management, especially in the stunning phase.

### 4.3 Relationship between Fractures and WHC Meat Quality

WHC was significantly associated with the occurrence of fractures—animals without fractures had higher WHC, regardless of the experimental group. This suggests an association between the physical integrity (absence of fractures) of the animals at the time of slaughter and the technological quality of the meat produced. The occurrence of fractures is often associated with preslaughter stress and excessive movement during electrical stunning.

Stress in pigs triggers complex physiological responses involving both the hypothalamic-pituitary-adrenal (HPA) axis—with the release of corticotropin-releasing hormone (CRH), adrenocorticotropic hormone (ACTH) and cortisol—and the sympathetic nervous system, responsible for the release of catecholamines, increased heart rate and redistribution of blood flow.^18^ Simultaneously, in the post-mortem period, there is an increase in glycolytic activity, which accelerates the degradation of muscle glycogen and promotes a sudden drop in pH, while the carcass temperature remains high. This condition favors the denaturation of proteins, reducing the water holding capacity of meat and negatively impacting its technological quality.^14^ Consequently, there is a higher incidence of PSE (pale, soft and exudative) meat.

Thus, the lower WHC observed in animals with fractures can be explained by the alteration in muscle metabolism induced by stress and trauma, favoring the production of meat with undesirable characteristics, such as PSE. This technological condition compromises the quality of the final product and generates losses to the industry: due to the greater loss of fluids during storage, or the shorter shelf life, or even the lower sensory acceptance.^14,18^

In a general basis, the results demonstrate that pork WHC was significantly associated with the occurrence of fractures, being higher in animals without fractures. This relationship was more evident in the magnesium-supplemented group (*p* = 0.0002), with the mean WHC being 35.71 for those without fractures and 33.32 for those with fractures. This result suggests that supplementation can improve meat quality, especially when there is no physical damage to the carcass.

Although magnesium supplementation did not directly alter the water holding capacity (WHC) in this experiment, the reduction in the occurrence of fractures can indirectly contribute to the maintenance of muscle integrity and meat quality, preventing economic losses associated with traumatic injuries.

## 5 Conclusion

The results of this research show that magnesium supplementation via drinking water in the hours prior slaughter can reduce the occurrence of vertebral fractures during electrical stunning of pigs, indicating a beneficial effect on the modulation of the response to acute preslaughter stress. The association observed between fractures and lower water holding capacity (WHC) reinforces the negative impact of these injuries on the technological quality of the meat. In addition, the relationship between lower thickness of subcutaneous fat and higher occurrence of fractures suggests that leaner animals may be more susceptible to these injuries.

This research has an innovative character by demonstrating, in a practical way, the potential of magnesium as a strategy of preslaughter water supplementation to improve animal welfare and preserve the quality of the final product, reducing economic and technological losses.

On the other hand, the research has limitations, such as the restricted number of parameters of meat aspects evaluated and the absence of physiological, biochemical and histological analyses that could elucidate the mechanisms involved. Future research should include different doses, durations and routes of administration of magnesium, in addition to the evaluation of physiological indicators such as (post-mortem pH, muscle glycogen, cortisol), technological (color, tenderness) and behavioral (observation protocols), to explore the understanding of its effects on the welfare and quality of pork.

## Abbreviations

WHC: water holding capacity
Ph: potential of hydrogen
*N*°: number
kg: kilogram
m^2^: square meter
C: control
T: treatment
L: liters
ppm: parts per million
UFU: federal university of uberlândia
OR: odds ratio
Amps: Ampere
Hz: hertz
°C: degrees celsius
cm^2^: square centimeters
ID: identification
CI: confidence interval
ANOVA: analysis of variance
CS: control with fracture
CN: control without fracture
TS: treatment with fracture
TN: treatment without fracture
PSE: pale, soft, and exudative
